# Platelets and Erythrocyte-Bound Platelets Bind Infectious HIV-1 in Plasma of Chronically Infected Patients

**DOI:** 10.1371/journal.pone.0081002

**Published:** 2013-11-25

**Authors:** Zoltan Beck, Linda L. Jagodzinski, Michael A. Eller, Doris Thelian, Gary R. Matyas, Anjali N. Kunz, Carl R. Alving

**Affiliations:** 1 U.S. Military HIV Research Program, Walter Reed Army Institute of Research, Silver Spring, Maryland, United States of America; 2 Henry M. Jackson Foundation for the Advancement of Military Medicine, Silver Spring, Maryland, United States of America; University of California, San Francisco, United States of America

## Abstract

Chronic HIV-1 infection is associated with persistent viremia in most patients, but it remains unclear how free virus may survive the potential hostile effects of plasma. We investigated whether sites might exist on the surfaces of circulating blood cells for protection of infectious HIV-1 particles. Red blood cells (RBC) either from blood of uninfected normal individuals, or from blood obtained without EDTA from chronically infected HIV-1 patients, invariably contained a small number of RBC having attached platelets as determined by flow cytometry, light microscopy, and immunofluorescence microscopy. After mixing normal RBC with platelet-rich plasma, discrete populations of RBC, platelets, and complexes of platelets attached to RBC were purified by fluorescence-activated cell sorting. Upon incubation of purified cells or platelets with HIV-1 followed by washing and co-incubation with CD4-positive peripheral blood mononuclear cells (PBMC), platelets, and platelet-RBC complexes, but not platelet-free RBC, caused infection of PBMC. Infection was prevented by pre-treating the platelet-RBC complexes with EDTA. Plasma and RBC (comprising a RBC/platelet-RBC mixture) from chronically infected patients with low viral loads were also co-incubated with PBMC *ex vivo* to determine the presence of infectious HIV-1. All freshly isolated plasmas from the HIV-1-infected donors, obtained in the absence of anticoagulant, were noninfectious. Interestingly, the RBC from most of the patients caused cell-cell infection of PBMC that was prevented by stripping the RBC with EDTA. A monoclonal antibody to DC-SIGN partially inhibited cell-cell HIV-1 infection of PBMC by normal RBC pre-incubated with platelets and HIV-1. We conclude: (a) platelet-free EDTA-free plasma from chronically infected HIV-1 patients, although containing viral RNA, is an environment that lacks detectable infectious HIV-1; (b) platelets and platelet-RBC complexes, but not purified RBC, bind infectious HIV-1; (c) DC-SIGN, and possibly other C-type lectins, may represent binding sites for infectious HIV-1 on platelets and platelet-RBC complexes.

## Introduction

After initial exposure to HIV-1 the human body wages a battle that leads to a standoff in which the virus remains chronically infectious within the host [1]. The appearance of HIV-1 RNA in blood plasma, defined as viral load, is generally thought to represent circulating cell-free virus particles that have the ability to infect new cells [2]. Antiretroviral therapy (ART) usually greatly suppresses HIV-1, reducing or even eliminating viral load and even causing HIV-1 to become latent such that the genetic imprint of the virus is harbored only within the genomes of infected cells. However, complete cure of HIV-1 infection is not achieved and non-latent reservoirs exist that cause low-levels of persistent viremia in most patients for many years [3], [4], [5], [6]. The extracellular environments of plasma and tissue fluids, which contain antibodies, complement, interferons, cytokines, enzymes, and various acute phase reactants and defensins, also represent potentially inhospitable environments faced by cell-free HIV-1 [1], [7], [8]. In support of this, plasma virus RNA levels, determined by quantitative competitive polymerase chain reaction methods, of 66 untreated or treated HIV-1-infected patients exceeded by an average of 60,000-fold the virus titers measured by endpoint dilution culture [2]. This suggested that most of the measured RNA was associated with noninfectious virus. Why is it so difficult to eradicate HIV-1 even in the face of both suppressive ART and numerous innate and adaptive anti-retroviral mechanisms? Although many theories have been proposed, immune and drug evasion hiding and sequestration strategies must exist leading to non-latent HIV-1 viruses.

One proposed mechanism that HIV-1 might use to avoid the potential dangers of plasma and other extracellular fluids is to undergo cell-cell transmission of virus [9], [10], [6], [11], [12]. The discovery of HIV-1 binding to the surfaces of uninfected dendritic cells (DC) via the C-type (calcium-binding) lectin family, of which DC-SIGN is an example, has helped to elucidate complex mechanisms of transmission of internalized and stored infectious HIV-1 that appears on the surfaces of uninfected DC for *trans* infection of T cells [10], [13], [14], [15], [16], [17]. In addition, it has been found that the surfaces of certain cells can serve as sanctuaries for infectious HIV-1, as illustrated by the observation that infectious HIV-1 apparently can persist on the surfaces of follicular dendritic cells for >9 months [18].

In considering possible mechanisms that might allow homeostatic maintenance of low level viremia we investigated whether infectious HIV-1 particles could find protection on the complex architectures of external surfaces of uninfected non-immune cells such as red blood cells (RBC). The existence of binding sites for infectious HIV-1 on the surfaces of RBC has been controversial. In support of this concept, *in vitro* binding of infectious HIV-1 to normal RBC was observed [19], [20], and was thought to represent a protective environment because inhibition by broadly neutralizing monoclonal antibodies of cell-cell infection of peripheral blood mononuclear cells (PBMC) by RBC-bound HIV-1 was reduced or absent [21]. The binding of HIV-1 protein or RNA to RBC obtained and studied *ex vivo* from HIV-1-infected patients was also reported [22], [23], [24]. However, in opposition, others denied the presence of viral RNA bound to RBC [25]. Still others proposed that binding of HIV-1 is restricted to RBC containing the blood group antigen known as Duffy receptor for chemokines [26], [27], [28], a conclusion that was disputed [29], [30], [31], [32]. In this study we have re-visited the question of RBC as a carrier of HIV-1. In addition, because HIV-1 has been reported to bind to and to be internalized by platelets (PLT) *in vitro* [33], [34], [35], [36], [37], [38], [39], [40], [41], [42], we investigated whether purified RBC might also contain some surface-attached PLT that could bind HIV-1 to cause cell-cell infection of PBMC.

## Results

### RBC obtained from HIV-1 infected or uninfected blood contain free and RBC-bound PLT

**Figure 1:**
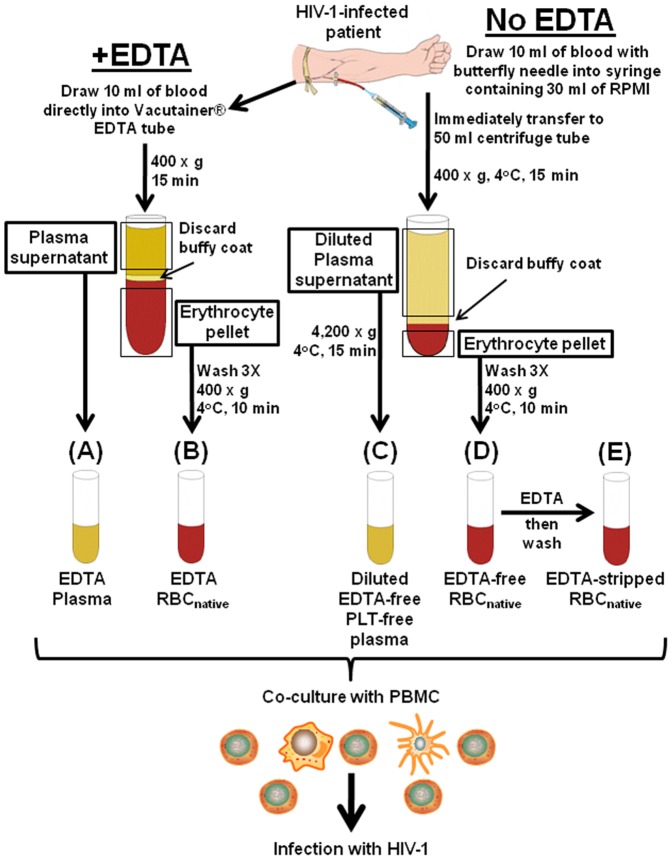
Fractionation of blood from HIV-1-infected patients for obtaining plasma and RBC_native_ in the presence or absence of EDTA. Whole blood collected from a patient in the presence (A,B) or absence (C,D) of EDTA, and separated into four plasma and erythrocyte fractions, A, B, C, and D, as shown. Fraction D was treated with 5 mM EDTA, resulting in Fraction E. All of the samples were used immediately for co-culture with PBMC to detect capability for causing HIV-1 infection of the PBMC. Aliquots were stored at −20°C for viral RNA measurements. See Materials and Methods for further details.

**Figure 2:**
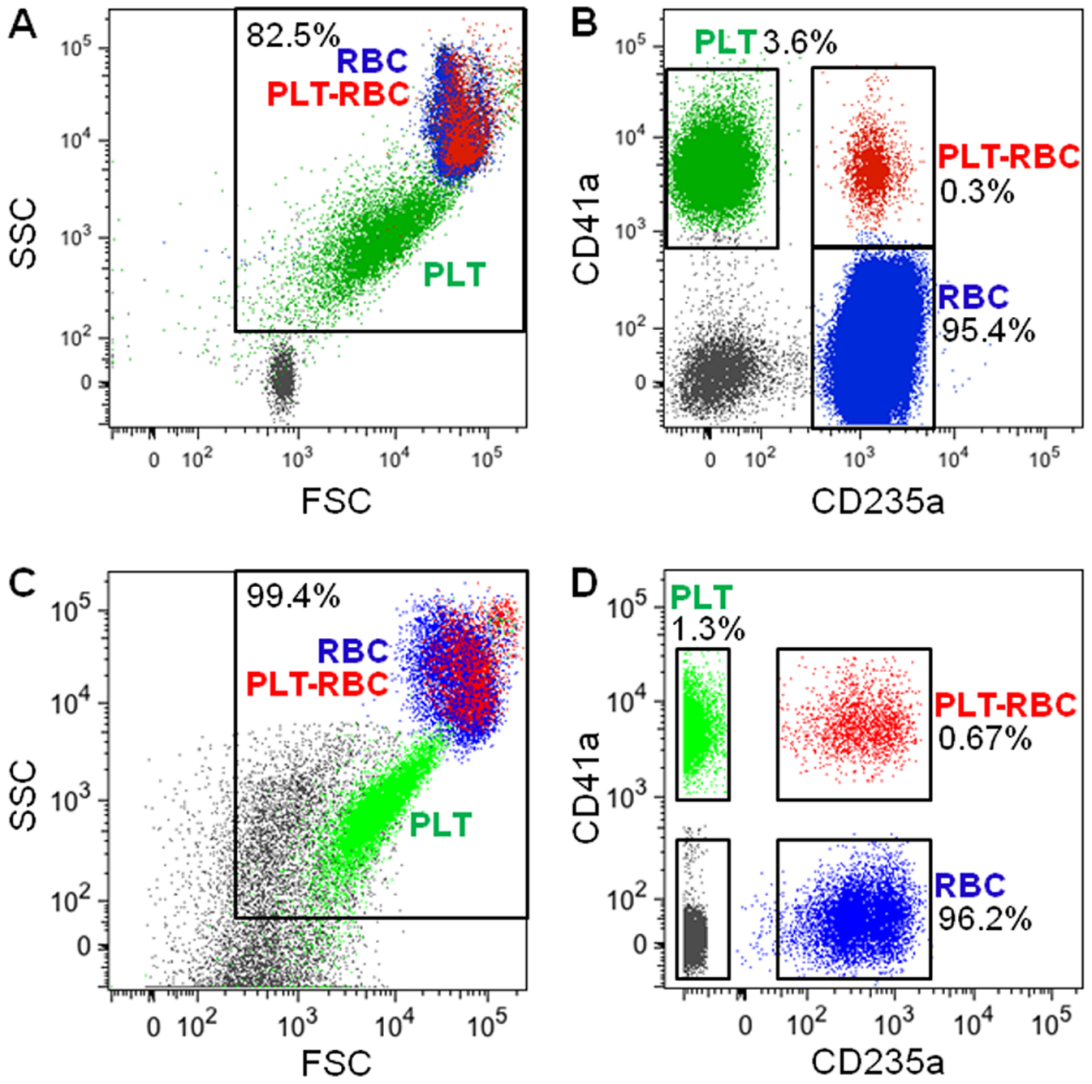
PLT-RBC is a subpopulation of cells in whole blood and in preparations of RBC_native_ from HIV-1-infected patients. A–B. Flow cytometry was performed with EDTA-anticoagulated whole blood obtained from an HIV-positive individual. A. Representative dot plot shows scatter properties of the RBC, PLT-RBC, and PLT. CD41a+/CD235a+ cells (red) represent PLT-RBC; CD41a+/CD235a- cells (green) represent free PLT; CD41a-/CD235a+ cells (blue) represent RBC; and CD41a-/CD235a- particles (gray) represent small vesicles or debris. B. Quantification of cells shown in frame A that express CD41a and/or CD235a. C–D. Flow cytometry was performed with an EDTA-free RBC_native_ preparation (see fraction D in Figure 1) obtained from an HIV-positive individual. RBC_native_ were analyzed as in frames A–B.

Whole EDTA-anticoagulated blood obtained from HIV-1-infected patients, or unsorted RBC preparations (RBC_native_) that were obtained in the absence of EDTA as shown in Fig 1D, were examined by flow cytometry after staining with antibodies to RBC (anti-CD235a) and antibodies to PLT (anti-CD41a) (Fig. 2A). In the EDTA-anticoagulated whole blood we observed cells that were dually stained with antibodies to both CD41a and CD235a (Fig. 2A), that comprised a small subpopulation (0.3%) of cells that consisted of PLT-RBC complexes (Fig. 2B). Among unsorted RBC preparations (RBC_native_) obtained from patients in the absence of EDTA (Fig. 1D) and examined for purity by flow cytometry, small amounts of contaminating free PLT were found, and were easily differentiated from the RBC because of the smaller size of the PLT (Fig. 2C). Detailed fluorescence-activated cell sorting (FACS) analysis further showed that three populations of cells were invariably present in the RBC_native_: RBC; a small number of contaminating free PLT; and a small stable population of PLT-RBC complexes (0.67%) (Fig. 2D).

**Figure 3:**
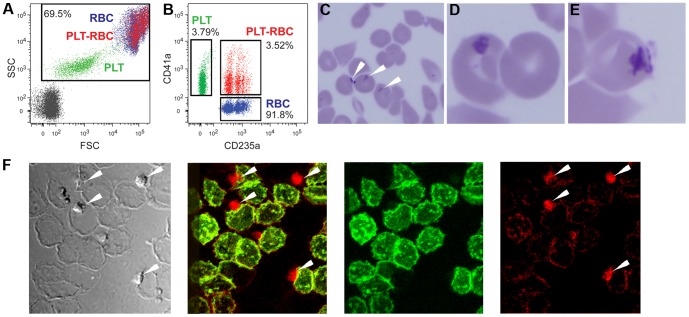
Enrichment of PLT-RBC as a subpopulation of normal (uninfected) cells by adding platelets to a preparation of RBC_native_ from a normal volunteer. A. Normal RBC_native_ obtained from citrated blood from a normal (uninfected) volunteer were incubated with platelet-rich plasma, washed, and analyzed by flow cytometry. B. RBC, PLT-RBC, and PLT were separated by sorting the populations shown in frame A. C–E. Light microscopy of Giemsa-stained mixtures of normal RBC_native_ enriched with PLT-RBC. Magnification: 400X and 1000X, respectively. Arrows in frame C indicate platelets bound to RBC. F. Fluorescent microscopy of PLT-RBC visualized at 630X magnification. Left panel, bright field; 2^nd^ panel, visualization of both FITC and PE signals; 3^rd^ panel, visualization of FITC; right panel, visualization of PE signal. Arrows point to platelets.

**Figure 4:**
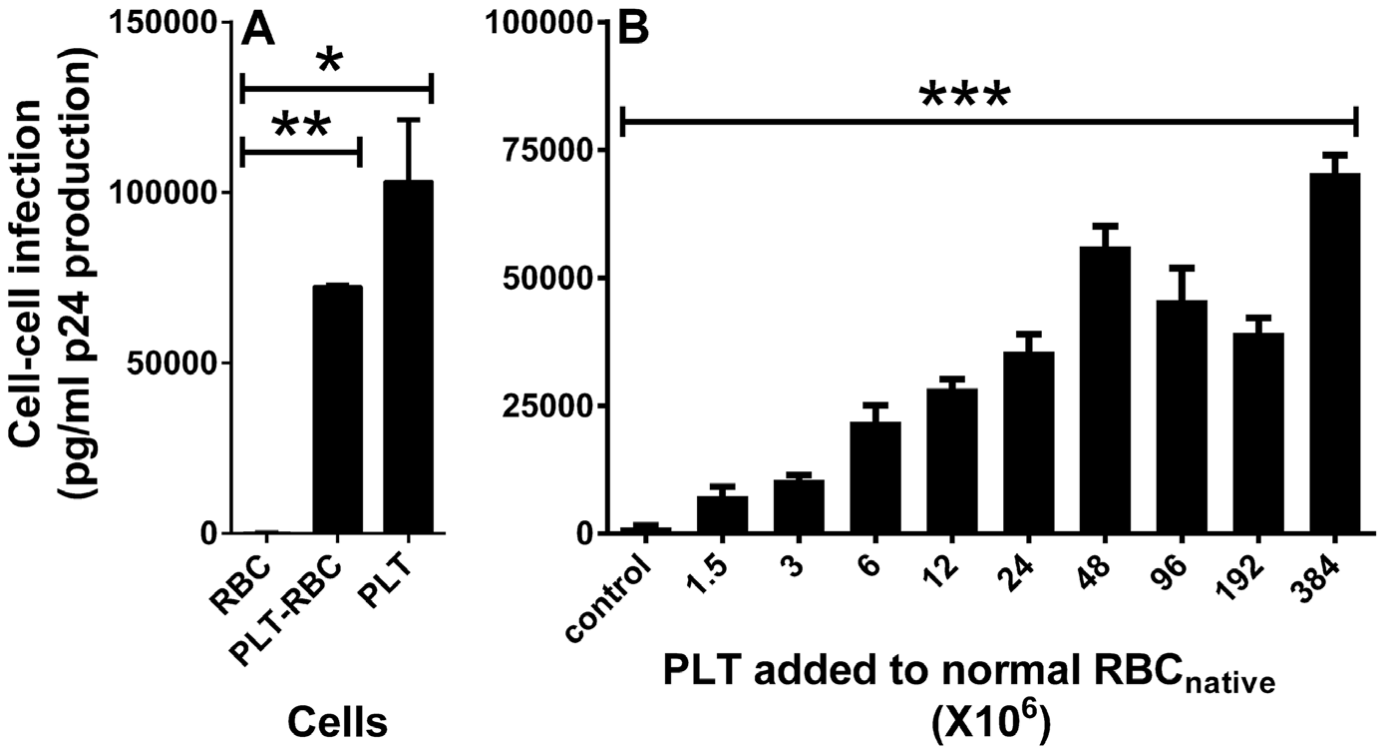
HIV-1 cell-cell infection of PBMC is dependent on PLT. **A**. RBC, PLT-RBC, and PLT were separated by sorting with FACS as shown in Fig. 3B. After incubation with HIV-1_Bal_, followed by washing 3 times to remove unbound HIV-1, the cells were co-incubated with PBMC to determine HIV-1 infection. **Cell-cell infection with PLT-bound HIV was higher than with RBC-bound HIV (p = 0.0332) (one-way Anova with Tukey's multiple comparisons test); ***cell-cell infection with PLT-RBC-bound HIV was higher than with RBC-bound HIV (p = 0.0124) (one-way ANOVA with Tukey's multiple comparisons test). **B**. Mixtures of normal RBC_native_ enriched with PLT-RBC were prepared by pre-incubation of 0.3 ml containing 10^9^ normal RBC_native_ with the indicated numbers of PLT in 0.3 ml RPMI dilutions of platelet-rich plasma, and then washed 3 times. The cells were then pre-incubated with HIV-1_Bal_, washed 3 times, and examined for HIV-1 infection of PBMC as described in Materials and Methods. Normal RBC_native_ not pre-incubated with PRP served as a control. *Cell-cell infection was increased as a function of the number of platelets added to RBC_native_ (p<0.0001, one way Anova).

**Figure 5:**
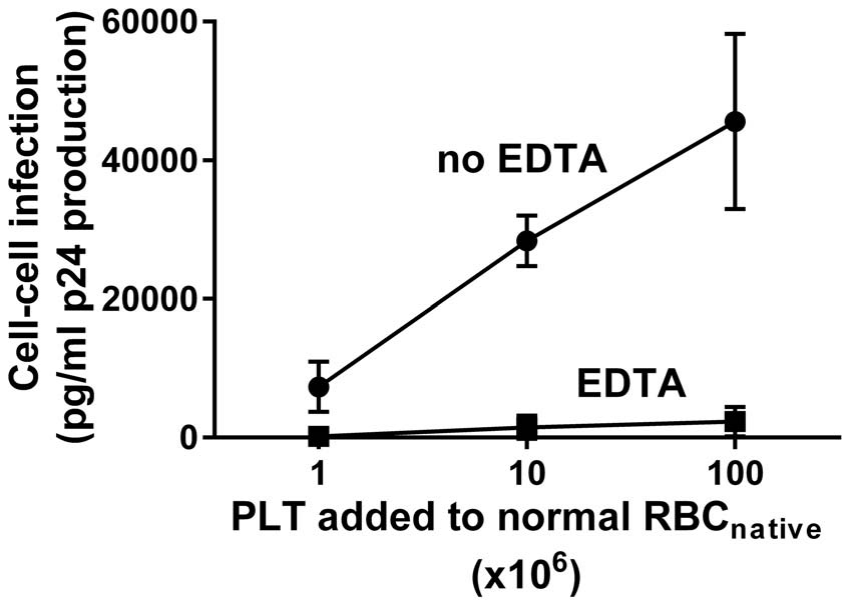
HIV-1 cell-cell infection of PBMC is eliminated by EDTA. PLT-RBC enriched RBC_native_ were prepared by pre-incubation of normal RBC_native_ (10^9^ cells) with the indicated numbers of PLT, washed 3 times, and pre-incubated with HIV-1_Bal_, washed 3 times, and then examined for HIV-1 infection of PBMC in the presence or absence of 5 mM EDTA. Cell-cell infection was eliminated by EDTA (p<0.0001, two-way Anova).

To model these three populations, normal (uninfected) RBC_native_ obtained from a commercial source from citrate-anticoagulated blood were incubated with PLT-rich plasma (PRP) and washed 3 times to try to remove contaminating cell-free PLT (Fig. 3A). As with the RBC_native_ from the infected patients, FACS analysis showed that the modeled normal RBC_native_ also contained RBC, PLT, and an enriched population of PLT-RBC (3.52%) (Fig. 3B). As described in the Methods, sometimes aggregates of PLT-RBC and RBC were observed, but these did not interfere with subsequent use of purified combined PLT-RBC or RBC fractions. Light microscopy (Fig. 3C–E) and immunofluorescent microscopy (Fig. 3F) of the modeled normal RBC_native_, revealed a distinct subpopulation of RBC that contained PLT-RBC complexes that could be easily visualized. Although many of the PLT attached to RBC had a normal discoid appearance (Fig 3D), many also appeared to be in an activated state (Fig. 3E). The normal PLT, RBC, and PLT-RBC were selectively sorted and purified by FACS (Fig. 3B), then pre-incubated with HIV-1, washed free of unbound HIV-1, and co-incubated with PBMC to examine their relative abilities to serve as carriers of HIV-1 for causing cell-cell infection of the PBMC. As shown in Fig. 4A, cell-cell infection of PBMC was supported by PLT or PLT-RBC but not by RBC alone. Pre-incubation of normal RBC_native_ with various dilutions of PRP to produce an enhanced subpopulation of PLT-RBC, followed by washing to remove unbound PLT, resulted in PLT-dose-dependent cell-cell infection (Fig. 4B). The infectivity was eliminated by treatment of modeled PLT-RBC with EDTA prior to incubation with PBMC (Fig. 5).

### Infectivity of plasma and RBC_native_ from HIV-1-infected patients

**Figure 6:**
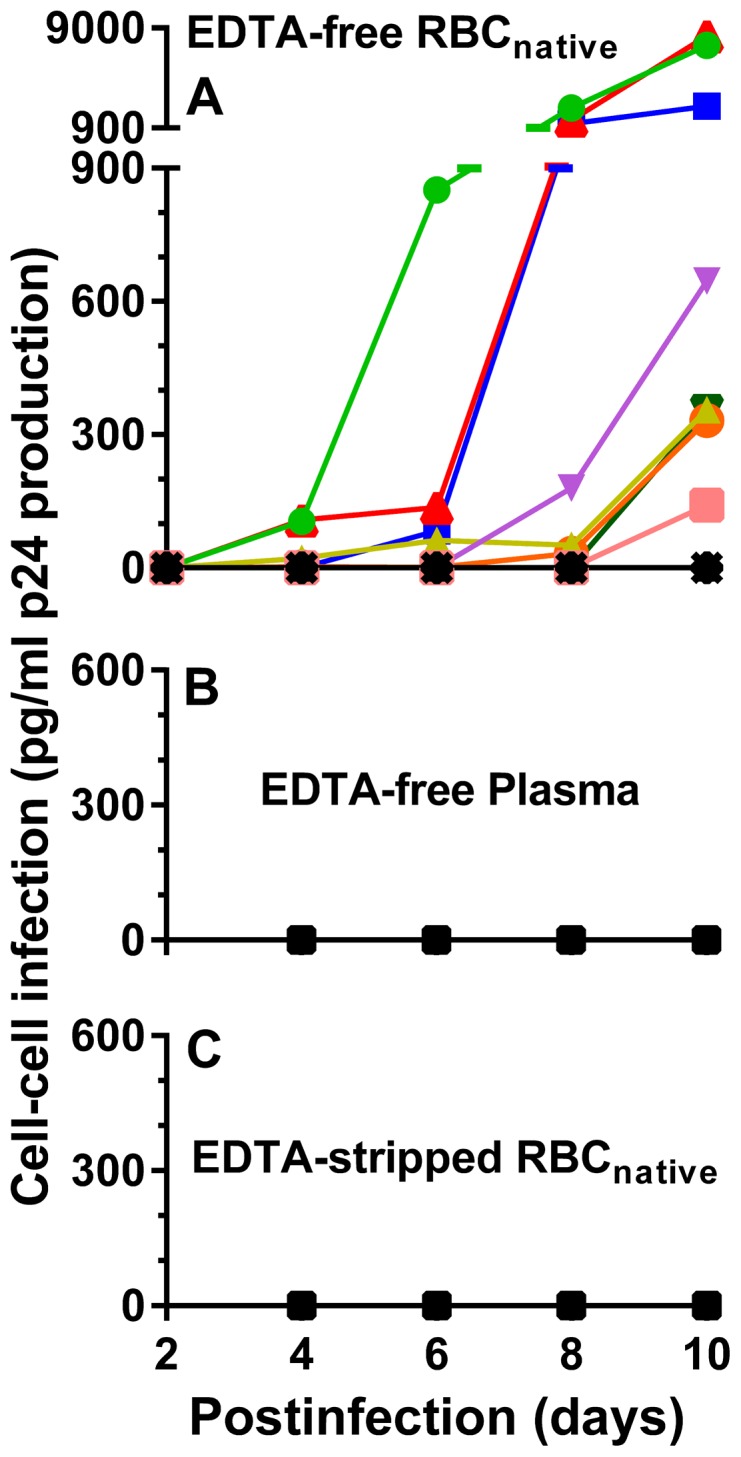
Cell-cell infection of PBMC by RBC_native_ from HIV-positive patients. **A**. EDTA-free RBC_native_ from 11 chronically-infected patients were obtained as in Fig. 1D and co-incubated with PBMC to examine infection of the PBMC. 25 µl of EDTA-free RBC_native_ were diluted in 75 µl of IL-2 medium and added to 50 µl of 1.5×10^5^ PHA-stimulated PBMC/well in IL-2 medium. **B**. 100 µl of EDTA-free plasmas from the patients, obtained as in Fig. 1C and supplemented with 20 U/ml of recombinant IL-2, did not infect co-incubated PBMC. **C**. The RBC_native_ shown in frame A were treated with 5 mM EDTA followed by washing 3 times in IL-2 medium before infecting PBMCs (see Fig. 1E) showed no HIV-1 infection of co-incubated PBMC. The infection exhibited by the group of EDTA-free RBC_native_ (**A**) was significantly higher using a paired t-test, than the infection exhibited both by the group of EDTA-free plasma (**B**) and by the group of EDTA-stripped RBC_native_ (**C**) at 8 days post-infection (p = 0.0404) and 10 days post-infection (p = 0.038).

The above results with modeled populations of PLT, PLT-RBC, and RBC *in vitro* suggested that a routine clinical preparation of RBC normally might also contain a small residual amount of PLT. However, even after complete removal of free PLT, the remaining RBC always contains a subpopulation of PLT-RBC, and it seems reasonable to hypothesize that the PLT and the subpopulation of PLT attached to RBC might each serve as an *in vivo* carrier of infectious HIV-1. To examine this with *ex vivo* cells, RBC_native_ (containing RBC and small subpopulations of PLT and PLT-RBC) were obtained from 11 chronically-infected HIV-1 patients having variable low levels of plasma viral load on initial screening as determined by RNA (Table 1). As shown in Table 1, 3 of the 11 patients were not on ART and 8 were on ART. EDTA-free RBC_native_ from the blood of the patients were obtained by rapid centrifugation of the blood in the absence of anticoagulant to prevent clotting [43] (Fig. 1D). All of the patients had detectable viral RNA in the initial screening plasma (Table 1), and 4 of the 11 plasmas subsequently lacked detectable RNA (Table 1, columns A and C). In contrast, all of the patients had detectable viral RNA both on the EDTA-RBC_native_ (Table 1, column B) and on the EDTA-free RBC_native_ (Table 1D). However, despite the frequent presence of HIV-1 RNA none of the EDTA-free plasmas from the patients (obtained as in Fig. 1C) caused infection of co-incubated PBMC (Fig. 6B). Furthermore, despite the presence of HIV-1 RNA none of the EDTA-RBC_native_ (obtained as in Fig. 1B), caused infection of co-incubated PBMC (data not shown), but 8 of 11 EDTA-free RBC_native_ (obtained as in Fig. 1D) caused infection of co-incubated PBMC (Fig. 6A). In every case the infection was prevented by pre-treatment of the RBC_native_ with EDTA (EDTA-stripped RBC_native_, as in Fig. 1E) (Fig. 6C). From these results it appeared that the PLT-RBC, and probably the free PLT, were highly infectious for transmission of virus to PBMC. However, the EDTA-free plasma, examined *ex vivo*, of every chronically infected HIV-1 patient was sterile with respect to infectious HIV-1 (Fig. 6B).

**Table 1:** HIV-1 RNA in plasma and RBC_native_ from chronically infected HIV-1 patients.

Patient no.	RNA copies/ml blood					
	Initial Screen	Experimental Plasma and RBC Fractions				
		(A)	(B)	(C)	(D)	
	EDTA-plasma^a^	EDTA-plasma^a^	EDTA-RBC_native_ b	EDTA-free plasma^c^	EDTA-free RBC_native_ d	Notes
1	5771	11610	2385	14784	3931	Diagnosis 2011 Never on ART
2	64814	4898	2059	4982	7668	Diagnosis 1984 ART since 1996
3	66	496	8106	177	9187	Diagnosis 1999 ART since 1999
4	98	306	4381	176	7706	Diagnosis 1996 ART since 1997
5	20	106	703	Not detected	2418	Diagnosis 1985 ART since 2000
6	72	Not detected	3439	Not detected	7925	Diagnosis 2006 Never on ART
7	252	Not detected	485	130	1345	Diagnosis 1997 ART since 1999
8	17108	42193	13471	42712	14309	Diagnosis 2009 Currently on ART
9	43	26	2421	80	693	Diagnosis 1996 Currently on ART
10	176	Not detected	317	Not detected	419	Diagnosis 1989 Currently on ART
11	613	Not detected	2542	Not detected	3536	Diagnosis 1990 Currently on ART

a,b,c,d Fractions were obtained as in Fig. 1A, 1B, 1C, and 1D, respectively.

### Inhibition of p24 binding and cell-cell infectivity of HIV-1 by a monoclonal antibody to DC-SIGN

**Figure 7:**
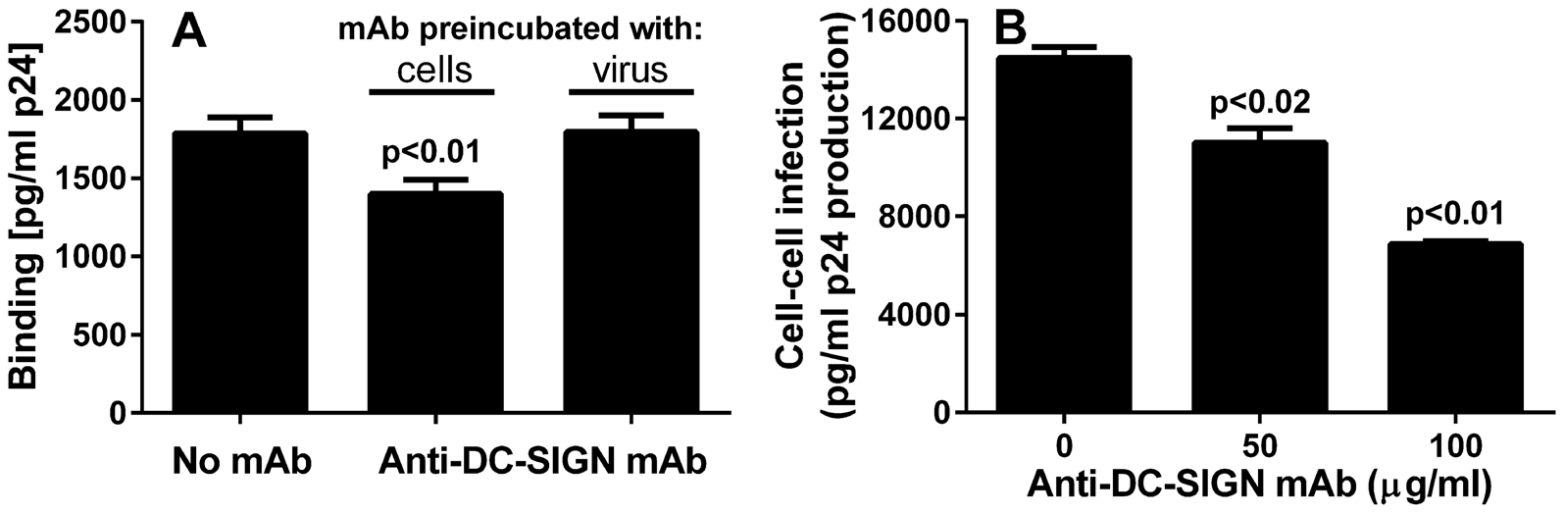
Binding of HIV-1 to RBC_native_ as well as cell-cell infection of PBMC is inhibited by anti-DC-SIGN monoclonal antibody. A. RBC_native_ was pre-incubated with HIV-1_Bal_ in the absence or presence of anti-DC-SIGN mAb in a final 50 µg/ml concentration, washed 3 times, and bound p24 was measured by ELISA. The binding was inhibited by pre-incubation of the cells with the monoclonal antibody (p<0.01, t-test), but not inhibited by pre-incubation of the virus with the monoclonal antibody. B. Cell-cell infection of PBMC was also inhibited in a dose-dependent manner by anti-DC-SIGN mAb (p<0.02 and p<0.01 respectively, paired t-test).

Inhibition by EDTA of cell-cell infectivity of normal RBC_native_ enriched with PLT and HIV-1, or by normal RBC_native_ from HIV-1-infected patients, raised the possibility that a PLT-associated C-type (calcium-binding) lectin, such as DC-SIGN [38], [39], [40], [41], might serve as a binding site for infectious HIV-1. Approximately 30% inhibition of binding of p24 to normal RBC_native_ was observed when an anti-DC-SIGN monoclonal antibody (mAb) was pre-incubated with normal RBC_native_; however no inhibition of binding was observed when the mAb was pre-incubated with HIV-1 (Fig. 7A). Pre-incubation of the anti-DC-SIGN mAb with normal RBC_native_ followed by addition of HIV-1 and co-incubation with PBMC resulted in a dose-dependent inhibition of cell-cell infectivity of the normal RBC_native_ (Fig. 7B).

## Discussion

Under optimal application of ART profound suppression or disappearance of detectable circulating viral RNA occurs in the blood of infected patients. In these circumstances it is believed that latent viruses exist that are reversibly nonproductive for infection, and the location and nature of these viral reservoirs remain controversial [5]. Among various non-latent viral reservoir maintenance mechanisms, the possibility has been proposed that persistence of low level infectious virus production from CD4-positive cells is still occurring, possibly even in circulating blood [3], [4], [5], [6]. Infection through cell-cell transfer of virus, which would not be expected to be detected as viremia, has been proposed as one possible mechanism for maintaining low levels of virus production even in the presence of ART [44]. Presumably, cell-cell infection through direct transfer of HIV-1 between infected and uninfected cells theoretically might protect the HIV-1 from direct exposure to some of the potentially hostile immunological and innate mediator effects that are present in extracellular fluids such as blood plasma [8], [11], [21]. Here we propose an evasion strategy for non-latent HIV-1 that consists of infectious HIV-1 persistence on the surface of non-infected PLT and PLT-RBC, which leads to infection associated with cell-surface-to-cell-surface transfer of infectious virions to CD4-positive cells that are permissive to infection. We suggest that PLT and PLT-RBC surfaces might be major sites for maintenance of the persistence of highly infectious HIV-1 in blood.

Our results are compatible with previous studies that suggested that HIV-1 can bind both to PLT and to megakaryocytes from which PLT are derived [33], [34], [35], [36], [37], [38], [39], [40], [41], [42]. We have further shown that a large number of PLT normally spontaneously attach to RBC, leading to PLT-RBC complexes. Previous studies have identified CXCL4 (also called platelet factor 4, or PF-4), derived from α-granules of activated degranulated platelets, as a “broad-spectrum” inhibitor of HIV-1 infection of T cells [45], [42]. The observations in the present study that both PLT and PLT-RBC support potent cell-cell HIV-1 infection of PBMC suggest that the PLT of each preparation either were not fully degranulated or that PLT-associated infectious HIV-1 overcame the inhibitory effects of CXCL4 released from those PLT that were degranulated.

The present results support previous reports that HIV-1 could bind to RBC preparations obtained from patients infected with HIV-1 [22], [23] [24] [27] [28], or to RBC preparations *in vitro* [19], [20], [21]. However, we now believe that in all of those previous studies that used RBC_native_ there were small subpopulations of PLT-RBC, or even residual free PLT, that were responsible for all of the binding of the virus. Thus, we conclude that infectious HIV-1 does not bind directly to RBC *per se*, but rather to PLT attached to RBC. In view of this, we believe that EDTA RBC_native_ obtained clinically might be a mixture of RBC and PLT-RBC, even if the cell-free PLT fraction is completely removed by differential centrifugation.

In considering a possible binding site for HIV-1 on PLT, the inhibition of cell-cell infection of PBMC by HIV-1 attached to PLT and PLT-RBC by EDTA (Fig. 5), and our ability to strip all of the infectious HIV-1 from the RBC_native_ of infected patients with EDTA (Fig. 5C), would seem to be a key observation. Several studies have reported that a fraction of PLT expresses the calcium-dependent lectin DC-SIGN which presumably could serve as a binding site for HIV-1 [38], [39], [40], [41], and our observation of partial inhibition of infection by antibodies to DC-SIGN is compatible with this possibility (Fig. 7). PLT also reportedly express another C-type (calcium-dependent) lectin, mannose receptor (MR), and also C-type lectin-like receptor 2 (CLEC-2) [39], [41], [42]. In view of this, DC-SIGN and possibly other members of the C-type lectin family, or CLEC-2, might represent binding sites for HIV-1 on PLT and PLT-RBC.

Regardless of the actual binding site for HIV-1 on RBC_native_, we have previously shown that the *in vitro* binding of the HIV-1 to RBC_native_ provided considerable protection against the antiviral effects of broadly neutralizing monoclonal antibodies [21]. Similar protection against monoclonal neutralizing antibodies was reported for HIV-1 bound to DC-SIGN on dendritic cells [46]. In view of this, we conclude that surface-to-surface transfer of infectious HIV-1 from PLT or PLT-RBC to CD4-positive cells might thus provide an immunologically protected environment that could permit survival and prolonged persistence of infectious virions that are produced in the presence or absence of ART.

The unexpected discovery in this study of the absence of infectious HIV-1 in the EDTA-free plasmas of all of the chronically infected patients having relatively low viral loads (Fig. 6B) suggests that the fluid phase of blood might constitute an inhospitable environment for HIV-1. Although all of the patients in the present cohort had relatively low viral loads, future studies will examine whether EDTA-free plasmas from patients having high viral loads are similarly sterile with respect to infectious HIV-1. The plasmas of most of the chronically infected patients in this study apparently were littered with what appeared to be corpses comprised of noninfectious incomplete virus containing RNA, or degradation products of HIV-1, a finding compatible with the 60,000-fold greater estimated virus levels detected by quantitative PCR when compared to endpoint dilution cultures [2]. When combined with the realization that the surfaces of PLT and PLT-RBC might be important reservoirs that harbor non-latent infectious HIV-1, these observations could have important theoretical implications for strategies of HIV-1 vaccine development. The well-known difficulty of inducing effective neutralizing antibodies to cell-free HIV-1 is widely viewed as a major impediment to vaccine development [47]. Cell-free infectious virus is the universal target used in laboratory-based neutralizing antibody assays [48], and we suggest that cell-free virus may be an inappropriate target for detecting effective neutralizing antibodies because plasma apparently might lack any infectious free virus. In fact, the possibility exists that the fluid phase of blood containing noninfectious HIV-1 particles might even serve as a sink for blocking neutralizing antibodies. Thus, the non-latent reservoir of cell surface-bound virus which is capable of cell-cell infection, might be more appropriate as a target for detection of clinically-relevant neutralizing antibodies.

## Materials and Methods

### Ethics statement

Blood was collected under WRAIR protocol #1749A (RV315A) entitled: Development of a Method to Obtain Human Erythrocytes without Anticoagulants from HIV Infected Volunteers and Assess Infectivity of the HIV Bound to the Erythrocytes, which is a substudy of WRAIR protocol #1749 (RV315) entitled, Collection and Processing of Blood from HIV Infected Donors for In Vitro Research. Both the parent protocol and the substudy were reviewed by the independent Institutional Review Board, Division of Human Subjects, Walter Reed Army Institute of Research. The parent study and the substudy were classified as minimal risk. HIV infected patient volunteers provided informed consent following discussion and counseling by the personnel at the WRAIR Clinical Trials Center. Investigators on this study were blinded to the identity of the volunteers. Volunteers were provided with a small stipend for participation in accordance with WRAIR and Federal policies, procedures, and guidance.

Peripheral blood samples from HIV-infected individuals were obtained under a protocol approved by the Institutional Review Boards of the Walter Reed Army Institute of Research and the Walter Reed National Military Medical Center, and the participants signed an informed consent document.

### Cells, Viruses, and Reagents

Primary HIV-1_Bal_ clade B isolate, was propagated and titrated in PBMC that were obtained by leukapheresis, and stored in cell culture supernatant containing RPMI 1640 (Quality Biologics, Gaithersburg, MD) with 15% heat-inactivated fetal bovine serum (Gemini Bioproducts, Woodland, CA) in liquid nitrogen as previously described [49]. Normal RBC_native_ and PRP, both collected with citrate-phosphate-dextrose-adenine anticoagulant, were purchased from Research Blood Components, Boston, MA. Normal RBC_native_ samples were washed in phenol red-free RPMI (Life Technologies, Carlsbad, CA) and stored for a maximum of three weeks in Adsol storage medium (1.1% dextrose monohydrate; 0.588% sodium citrate dihydrate; 0.41% NaCl; 0.276% monobasic sodium phosphate monohydrate; 0.042% citric acid monohydrate; 0.03% adenine) at 4°C. Before use, RBC_native_ were quantified by Coulter Analyzer ACT10 (Beckman Coulter, Fullerton, CA); then washed with phenol red-free RPMI and re-suspended in IL-2 medium consisting of RPMI 1640 with 1% penicillin-streptomycin and L-glutamine (both from Quality Biologics), 15% fetal bovine serum, and 20 U/ml recombinant IL-2 (Roche, Indianapolis, IN). Infection of PBMC was carried out in IL-2 medium. Infection was quantified by p24 production measured with a p24 antigen capture ELISA kit (Advanced BioScience Laboratories, Kensington, MD). Based on standard curve measurements, the detection limit for p24 was 2.5 pg of p24/ml. Mouse monoclonal antibody to human DC-SIGN (MAB161) was purchased from R&D Systems (Minneapolis, MN).

### Preparation of plasma and RBC_native_ from infected patients

Blood samples from 11 walk-in patients having detectable plasma HIV-1 RNA at the screening visit were collected at the Rockville Vaccine Assessment Clinic or Clinical Trials Center of the Walter Reed Army Institute of Research. As shown in Table 1, although all of the patient volunteers were chronically infected, they were heterogeneous with respect to length of infection, viral RNA load, and status of ART. Whole blood collected in the presence or absence of EDTA was separated into four plasma and erythrocyte fractions by rapid centrifugation, as shown in Fig. 1(A)–1(D). Centrifugation of whole blood was performed within 10 min in the absence of EDTA as described previously [43]. This would not be expected to induce activation and increased stickiness of platelets in view of the observation that platelet-derived microvesicles, a sign of platelet activation, are not increased in EDTA-free plasma obtained using this method [43]. Fractions comprising plasma fraction A, and erythrocytes fraction B, were obtained in the presence of EDTA (Fig. 1). Fractions comprising plasma fraction C, and erythrocytes fraction D, were obtained in the absence of EDTA using the methods shown in Fig. 1. Because of initial dilution of blood in the absence of EDTA the plasma comprising fraction (C) was diluted when compared with fraction (A). Fraction (E) was obtained by adding EDTA in a final concentration of 5 mM to fraction (D) at 4°C for 30 min, and the resulting EDTA-stripped RBC were then washed three times in phenol red-free RPMI and re-suspended in IL-2 medium. All of the samples were used immediately for PBMC infection and aliquots were stored at −20°C for viral RNA measurements.

### Preparation of normal RBC_native_ enriched with PLT-RBC

One ml of normal RBC_native_ (2.5×10^9^) in RPMI was incubated with 0.3 ml of PRP dilutions in RPMI for 1 hour at room temperature with rotation. Cells were washed 3 times in phenol red-free RPMI, and unbound platelets were removed by centrifugation at 400× g to produce a mixture of RBC_native_ enriched with PLT-RBC and re-suspended at 2.5×10^9^ cells/ml in IL-2 medium.

### Attachment of HIV_Bal_ to normal RBC, PLT, and PLT-RBC

Sorted cells or PLT were concentrated to 5×10^7^/ml in phenol red-free RPMI, and incubated with an equal volume of HIV_Bal_ (containing 148 ng of p24/ml) for 1 hour at room temperature with slow rotation. After washing 3 times to remove unbound HIV, the PLT, RBC, or PLT-RBC-bound viruses (2.5×10^6^ of PLT, RBC, or PLT-RBC in 50 µl IL-2 medium) were co-incubated with PHA-stimulated PBMC, as described below.

### Infection and cell-cell infection of PBMC

Infection of PBMC by HIV-1_bal_ attached to sorted RBC, PLT-RBC, or PLT, or by plasma or RBC_native_ obtained from HIV-1-infected patients, was performed as modified from Brown et al [49]. In short, indicated volumes or numbers of plasma or RBC, respectively, in IL-2 medium were incubated with 1.5×10^5^ of PHA-stimulated PBMC. In each case, 70 µl of fresh IL-2 medium were added to the cells after 24 hours. On days 3, 4, 6, 8, and 10 post-infection, 50 µl of culture fluid was harvested and assayed for HIV-1 p24, and 50 µl of IL-2 medium was added back to the culture, as needed for continued incubation.

### Flow cytometry and FACS

For flow cytometry and FACS studies the staining buffer contained 0.5% bovine serum albumin and 20 mM HEPES in phenol-free RPMI, and the staining cocktail consisted of CD235a (clone 10F7MN) conjugated to fluorescein isothiocyanate (FITC) (eBiosciences, San Diego, CA), CD4 (clone T4) conjugated to energy coupled dye (ECD) (Beckman Coulter, Fullerton, CA), CD45 (clone 2D1) conjugated to peridinin chlorophyll protein, and CD41a (clone HIP8) conjugated to allophycocyanin (APC) (BD BioSciences, San Jose, CA).

Antibodies to CD235a (a highly negatively charged-sialoglycoprotein) can cause massive aggregation of RBCs. While some of this is no doubt due to cross-linking by bivalent antibodies, it can be exacerbated when uncharged CD235a-PE conjugates are utilized. When the same CD235a clone is used in fluorescein-conjugated form (fluorescein is negatively charged), higher concentrations of antibody can be used without risk of causing massive RBC aggregation (D. R. Sutherland, personal communication) [50]. Based on this we used clones that greatly reduce charge masking and reduce aggregate formation. In addition, we used methods that aggregate formation, particularly when staining RBC for flow cytometry, such as: titering down antibodies, using directly conjugated antibodies, staining at room temperature, logarithmic scaling for forward scatter, side scatter, thorough vortexing and mixing of samples, and fixing samples with formaldehyde to reduce agglutination [51]. Together, these procedures significantly reduced aggregate formation for the phenotyping of RBC and PLT blood product. This procedure was also used for sorting experiments. However, because the experiments were conducted without fixation and on a significantly scaled up procedure, some aggregates were observed during the flow acquisition on the FACS Aria SORP. Despite some observed aggregates, distinct populations of PLT, RBC-PLT, and RBC were obtained that were suitable for cell-cell infection experiments.

Normal RBC_native_ enriched with PLT-RBC, or RBC_native_ obtained from HIV-positive patients, were transferred to 96 well polypropylene plates and diluted in staining buffer, centrifuged at 300× g for 6 minutes, and the cells were re-suspended with 10% normal mouse IgG in staining buffer and incubated in the dark for 15 minutes at room temperature. They were then centrifuged at 300× g for 6 minutes and re-suspended in staining cocktail for 30 minutes. Cells were washed twice and re-suspended in 2% formaldehyde for 15 minutes. Samples were washed twice and re-suspended in staining buffer before acquisition on a four laser LSR II (50 mW 488 nm, 100 mW 405 nm, 40 mW 639 nm, and 150 mW 532 nm) with DIVA software version 6.2 (BD BioSciences). All flow cytometry analysis was performed using Flow Jo software, version 9.5.3 (Treestar Inc, Ashland, OR).

For purification of individual fractions by FACS, mixtures of normal RBC_native_ enriched with PLT-RBC were diluted to a concentration of 10^9^ RBC/ml in staining buffer. After centrifugation at 800× g for 10 minutes, samples were re-suspended in 10% human serum in staining buffer to block non-specific Fc receptor binding, and incubated in the dark for 15 minutes at room temperature. Samples were centrifuged at 800× g for 10 minutes, supernatants were discarded and the samples were re-suspended in staining cocktail for 30 minutes. Cells were washed twice with staining buffer and re-suspended at a final concentration of 10^7^ cells/ml for sorting. A four laser FACS Aria SORP (100 mW 488 nm, 100 mW 405 nm, 40 mW 639 nm, and 150 mW 532 nm) with DIVA software was used to sort into PLT, RBC, PLT-RBC populations for subsequent viral *trans* infection experiments.

### Fluorescence Microscopy

Normal RBC_native_ enriched with PLT-RBC were fixed with paraformaldehyde and blocked with 10% goat serum. Cells were stained with mouse anti-CD235a (clone JC159; Dako, Carpinteria, CA) and rabbit anti-CD41a (Abcam, Cambridge, MA) primary antibodies and goat polyclonal anti-mouse-FITC and goat polyclonal anti-rabbit-PE secondary antibodies, and imaged on a Zeiss 710 confocal microscope. Isotype controls consisted of nonspecific mouse IgG_1_ (BD Biosciences, San Jose, CA) and rabbit IgG antibodies (Abcam) The FITC was excited at 488 nm and the collected wavelengths of light were from 500–550 nm. The PE was excited at 561 nm and the collected wavelengths were from 570–620 nm. These images were collected using a 63X oil immersion objective with the confocal aperture set at 1 Airy unit. The z-stack interval was 0.4 micron. For the fluorescence channels, image stacks were flattened to a single image using a maximum intensity projection. For the bright field image the differential interference contrast channel was used.

### HIV-1 RNA measurement

Cell-free HIV-1 RNA was quantified in EDTA and RPMI-diluted plasma samples according to the manufacturer's instruction using the Roche COBAS AmpliPrep/COBAS TaqMan HIV-1 v2.0 (TaqMan HIV-1) test with minor modifications. RPMI-diluted plasma specimens were clarified of any cellular material by centrifugation at 3000× g for ten minutes and tested per standard operating procedures. Plasma specimens having less than 1 ml were diluted in SPEX lysis buffer (Roche Molecular Diagnostics, Inc., Indianapolis, IN) in order to have sufficient volume for testing. HIV-1 RNA was quantified in RBC_native_ exposed to EDTA and EDTA-free RBC_native_ preparations using the same TaqMan HIV-1 test with minor modifications. To avoid inhibition and clotting, a pre-extraction procedure was performed for RBC_native_ using the Qiagen DNA Blood Mini Extraction kit (Qiagen, Valencia, CA) with the inclusion of an additional wash at each washing step to ensure removal of hemoglobin. The eluted Qiagen extract was diluted into SPEX for testing in the TaqMan HIV-1 test. Values obtained from HIV-1-spiked plasma specimens tested directly on the TaqMan HIV-1 test were comparable to pre-extracted TaqMan HIV-1-tested specimens. HIV-1 RNA test values were adjusted for dilution and volume, if applicable. RNA copies per fraction were calculated from the total volume for each specimen. The percentage of HIV-1 RNA per fraction was calculated by dividing the total RNA in each fraction (plasma or RBC_native_) by the sum of RNA copies in the plasma and RBC_native_ fractions.

### Statistics

Statistical analyses were conducted using GraphPad Prism. Data were analyzed using one-way Anova, one-way Anova with Tukey's correction for multiple comparisons, two-way Anova, or paired t-test as indicated in the figures.
